# Sustained release local anesthetics for pain management: relevance and formulation approaches

**DOI:** 10.3389/fpain.2024.1383461

**Published:** 2024-04-05

**Authors:** Melese Getachew, Hana Tesfaye, Wubetu Yihunie, Tesfahun Ayenew, Sintayehu Alemu, Ephrem Mebratu Dagnew, Yalemgeta Biyazin, Dehnnet Abebe, Natanim Degefu, Abtie Abebaw

**Affiliations:** ^1^ Department of Pharmacy, College of Medicine and Health Sciences, Debre Markos University, Debre Markos, Ethiopia; ^2^ School of Pharmacy, College of Medicine and Health Sciences, University of Gondar, Gondar, Ethiopia; ^3^ Department of Nursing, College of Medicine and Health Sciences, Debre Markos University, Debre Markos, Ethiopia; ^4^ Department of Pharmaceutics, School of Pharmacy, Institute of Health, Jimma University, Jimma, Ethiopia; ^5^ Department of Pediatrics and Child Health Nursing, College of Medicine and Health Sciences, Debre Markos University, Debre Markos, Ethiopia; ^6^ Department of Pharmaceutics, School of Pharmacy, College of Health and Medical Sciences, Haramaya University, Harar, Ethiopia; ^7^ Department of Medical Laboratory Sciences, College of Medicine and Health Sciences, Debre Markos University, Debre Markos, Ethiopia

**Keywords:** chronic pain, formulation approaches, liposomal formulations, lipid nanoparticles, local anesthetics, localized analgesia, polymeric matrices, regional analgesia

## Abstract

This review attempted to ascertain the rationale for the formulation of sustained-release local anesthetics and summarize the various formulation approaches designed to date to achieve sustained and localized local analgesic effects. The incidence of pain, which is the concern of patients as well as health care professionals, is increasing due to accidents, surgical procedures, and other diseases. Local anesthetics can be used for the management of moderate to severe acute and chronic pain. They also allow regional analgesia, in situations where the cause and source of the pain are limited to a particular site or region, without the need for loss of consciousness or systemic administration of other analgesics thereby decreasing the risk of potential toxicities. Though they have an interesting antipain efficacy, the short duration of action of local anesthetics makes the need for their multiple injections or opioid adjuvants mandatory. To overcome this problem, different formulations are being designed that help achieve prolonged analgesia with a single dose of administration. Combination with adjuvants, liposomal formulations, lipid-based nanoparticles, thermo-responsive nanogels, microspheres, microcapsules, complexation with multivalent counterions and HP-β-CD, lipid-based nanoparticles, and bio-adhesive films, and polymeric matrices are among the approaches. Further safety studies are required to ensure the safe and effective utilization of sustained-release local anesthetics. Moreover, the release kinetics of the various formulations should be adequately established.

## 1 Background

Pain is an unpleasant sensory and emotional experience associated with, or resembling that associated with, actual or potential tissue damage (1). Nociception on the other hand is defined as the neural process that encodes and processes noxious stimuli, activating sensory receptors, transmitting signals, and detecting pain, crucial for survival and injury protection (2). It arises due to trauma (accident or surgical procedures associated) or various diseases thet makes the top list of complaints presented complaints to physicians. Relieving pain has been shown to result in improved healing, faster recovery, and an earlier return to former activities and lifestyle. Hence, pain management has become a prominent issue for healthcare practitioners and patients. The control of pain should be one of the major components of treatment goals whether the medical interventions could/not cure diseases to ensure patient comfort (3, 4).

Extracts of Erythroxylon coca have long been used to produce analgesia and euphoria for centuries by the indigenous population of Peru, they called this plant “khoka” as a reflection of its importance in their economy. Following the conquest of Peru by Francisco Pizzaro after 1530, the leaves of this plant have been used by ancient civilizations such as Sumerians, Greece, and the Roman Empire as analgesia and euphoric agents for centuries (5). Niemann, a young PhD student in Germany, isolated the first local anesthetic cocaine (an alkaloid) from coca plant leaves by the year 1860. In due course and following some experimentation with colleagues, Koller described the first clinical use of a local anesthetic by applying cocaine topically to facilitate glaucoma surgery in 1884 (3, 6).

Since Koller's report in 1884, local anesthetics have been used clinically for the management of acute or chronic pain conditions including post-surgical pain. Gordh was the first to use the amide drug, lignocaine (in 1948); the amide local anesthetics are used now in preference to the esters as they have fewer undesirable effects. About the mid of the 20th century, procaine (Novocain) was synthesized followed by lidocaine (1943), mepivacaine (1956), bupivacaine (1963), and ropivacaine (1996); all agents still in use today (6).

Though local anesthetics advanced pain management to a less toxic and costly regimen compared with the use of opioid analgesics, their short duration of action, which ranges from minutes to not more than four hours, became the major area of concern (7). Moreover, the risk of systemic toxicity and adverse local tissue reactions are common with high doses of local anesthetics (8). Different approaches have been to prolong the duration of action of local anesthetics; including a combination with adjuvants, liposomes, microemulsions, microspheres and microcrystals, complexation, nanoparticles, polymeric matrices, bioadhesive films, and lipid-protein-sugar particles.

This review aimed to depict the rationale behind the formulation of sustained-release local anesthetics and summarize the various formulation approaches that have been attempted to date.

## 2 Main text

### 2.1 Role of local anesthetics in pain management

Nearly 313 million surgical procedures are estimated to be performed each year globally. Unfortunately, as much as 80% of these patients experience moderate-to-severe acute pain after surgery. Moreover, 10%–60% of such patients are reported to develop chronic pain (4, 9). Inadequate management of surgical pain can delay surgical recovery, decrease patient satisfaction, and increase the length of hospitalization, readmission rates, and overall healthcare costs. The adequacy and suitability of postoperative pain control is also one of the most important factors in determining when a patient can be safely discharged from the inpatient facility. Hence, the availability and choice of an appropriate analgesic should be of great concern to ensure effective and safe means of pain management (9, 10).

Opioids have long been used to control pain in patients, including surgery-associated peri- and post-operative pain. However, the use of opioid analgesics for pain management has numerous side effects called opioid-related adverse drug events. Respiratory depression is one of the most potentially serious as it can be life-threatening. On the other hand, ileus is one of the most troublesome side effect that contributes to considerable patient discomfort and delayed discharge. These opioid-related adverse drug events can have a considerable impact on patient recovery after surgery and contribute to the clinical and economic impact of postsurgical care (5, 10).

The use of multimodal analgesic regimens is a practical way to achieve good postsurgical analgesia while minimizing reliance on opioids and associated adverse events. Peripheral nerve blocks and wound infiltration with local anesthetics are commonly used techniques because they can provide effective intra- and postoperative analgesia. The infiltration of wounds with local anesthetics not only provides analgesia but also appears to reduce the local inflammatory response to trauma or surgery. This in turn may help reduce the upregulation of peripheral nociceptors that manifests as hypersensitivity to a stimulus. As a result, these techniques can decrease the anesthetic and analgesic requirements during surgery and reduce the need for opioid analgesics in the postoperative period. More effective pain relief in the early postoperative period from the residual sensory block provided by local anesthesia can facilitate the recovery process, enabling earlier ambulation and discharge to home (11, 12).

### 2.2 The need for sustained release local anesthetics

Surgery is ever changing its basis from an inpatient to outpatient setting because of technological advances and many other reasons such as patient preference and the high cost associated with admission. About 70% of all surgical procedures are carried out outside of a hospital setting in the United States. One of the elements determining whether the procedure will be performed on an outpatient basis or if admission and a hospital stay are required is pain management (13). In clinical practice, pain is one of the most frequent causes of unscheduled hospital admission or readmission. It is now understood that issues linked to anesthetic, rather than surgical factors, affect the decision to perform more invasive procedures as outpatients. Hence, effective and safe control of moderate to severe pain for the unmonitored patients at home for several days is critical. Local anesthesia will serve as a mainstay of postoperative pain control for both effective outpatient surgery and for discharged patients (14, 15).

Despite their widespread use in the treatment of both acute and chronic pain, local anesthetics' use was limited because of their short duration of action. Catheter infusions and repeated injections have been used to achieve long and persistent pain relief (16). Postoperative pain can effectively be relieved by continuous infusion of local anesthetics into the surgical wound and this technique provides good analgesia with less morphine consumption and decreased risk of adverse effects (7). As a result of technological improvements that make the insertion and maintenance of peripheral nerve catheters more dependable and safer, they are frequently employed in both the hospital and the outpatient setting. The flexibility of a catheter is one benefit it has over a single injection technique in that the infusion can be stopped, increased, or lowered at any time (17, 18). However, a peripheral nerve catheter is not necessary for all individuals to manage their postoperative pain, though. Moreover, not every anesthesiologist or practice situation will benefit from these devices. Catheter procedures are typically time-consuming, labor-intensive, awkward, and expensive (5, 18, 19). Other shortcomings of this technique include the high price of the infusion device, the need for hospitalization, the risk of infection, and occasionally irreparable muscle injury (13, 20).

Therefore, it is obvious that a single injection of a long-lasting local anesthetic that is safely administered and offers a predictable time of relief with barely any motor blockage could be appealing to patients. In the ideal scenario, anesthesia would provide a quick and effective way to deliver continuous postoperative pain relief without incurring a substantial cost in terms of training, people, time, and expensive institutional assets. Long-acting local anesthetics will be the best alternatives in this situation and the simple and familiar steps involved in performing a peripheral nerve block remain unaltered (21, 22).

### 2.3 Formulation approaches for sustained release local anesthetics

Conventional local anesthetic administration does not, usually, provide prolonged or localized drug release to specific targets. In many cases, these products provide a short period of analgesia with an increased risk of toxicity due to a higher extent of systemic absorptions. Following a relatively short period at the therapeutic level, drug concentration eventually drops off till the administration of the next dose.

**Figure 1:**
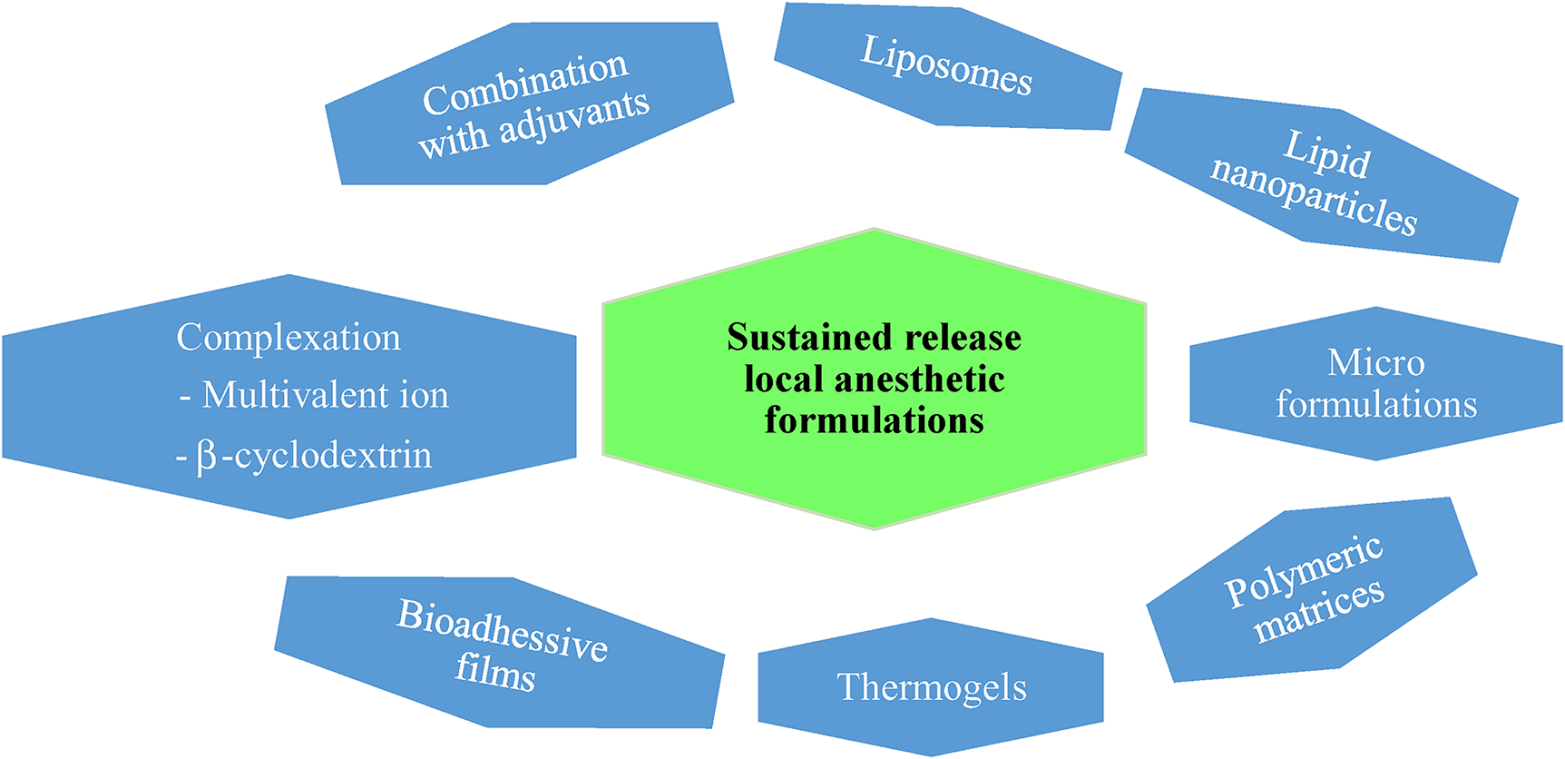
Schematic representation of the various formulations included in the review.

Hence, new formulation approaches are getting attention to achieve rate-controlled and prolonged release of local anesthetics. Different formulation approaches have been designed to achieve this goal. These include combination with adjuvants, liposomes, micro- and nano formulations, thermogels, multivalent ion and polymer comolexes, and bioadhesives (Figure 1). Each formulation offers unique advantages in terms of drug delivery and therapeutic outcomes but also comes with potential drawbacks that need to be considered during formulation development and clinical practice. A summary of the basic features and pros and cons of the various formulation approaches discussed in this review is presented in Table 1.

**Table 1:** Descriptions of various sustained-release local anesthetic formulations.

Formulation	Basic features	Formulated LA/s	Advantages	Disadvantages
Combination with Adjuvants	The local anesthetic is formulated along with a variety of agents with varying activity	Lidocaine (23, 24)	Increased efficacy Prolonged duration of action Reduced systemic toxicity Enhanced analgesic effect	Potential for drug interactions Increased risk of adverse reactions Complex formulation requirements
Liposomes	Liposomes encapsulate the local anesthetic, prolonging its release.	Bupivacaine (22, 25, 26) Ropivacaine (27)	Improved drug stability Controlled release profile Enhanced drug penetration into tissues Decreased systemic toxicity	Complex manufacturing process Potential of leakage
Lipid-based nanoparticles	Nanoparticles or nanofibers enable controlled release of the local anesthetic	Lidocaine (21) Ropivacaine (28) Bupivacaine (29, 30)	Enhanced drug solubility Improved bioavailability Targeted drug delivery	Risk of lipid oxidation Potential for particle aggregation, Limited drug loading capacity
Polymeric matrices	Implants implanted near the site of action continuously release the anesthetic.	Levobupivacaine & Lidocaine (31) Bupivacaine (32) Ropivacaine (33, 34)	Prolonged and controlled drug release. Reduced dosing frequency	Surgical insertion required Potential for infection at the implant site
Thermogels	Temperature-sensitive gelling and drug release	Lidocaine (16, 35–37) Ropivacaine (38, 39) Benzocaine (40)	Ease administration Site-specific delivery Tunable gelation properties	Limited thermal stability Potential for gel degradation in the body Complex formulation requirements
Microspheres and Microcapsules	Microparticles slowly release the local anesthetic over time.	Dibucaine (41) Tetracaine (42) Bupivacaine (43, 44)	Sustained release of drug Reduced frequency of dosing Protection of the drug from degradation	Batch-to-batch variability Potential for burst release Variability in drug release kinetics
Multivalent-ion complexation	A mild initial burst followed by prolonged of the local anesthetic	Lidocaine (3)	No need for polymers	Toxicity may arise from salts used
Complexation with cyclodextrin	Absorption into systemic circulation is slowed because of the hydrophilic nature of the complexing agent	Tetracaine (45) Bupivacaine (46) Ropivacaine (46–48) Benzocaine (49) Lidocaine (50)	Enhanced drug solubility Improved stability Increased bioavailability	Risk of complex dissociation Limited applicability to certain drugs Potential for off-target effects
Bio-adhesive films		Benzocaine (46) Lidocaine (51, 52) Lidocaine and ropivacaine (53, 54)	Prolonged contact time at the application site Enhanced drug retention Improved patient compliance	Variable adhesion properties Risk of film detachment Limited flexibility in dosing regimens

#### 2.3.1 Combination with adjuvants

Increasing the duration of local anesthetic action is often desirable because it prolongs surgical anesthesia and analgesia. In some clinical settings, it may be necessary to inject large volumes (consequently very high doses) of local anesthetics to provide an adequate level of block. Subsequently, these high doses have led to systemic toxicity due to the increased rate and extent of absorption of the medicament into the systemic circulation. Different additives with varying mechanisms of action have been used to prolong regional nerve blockade. Systemic absorption of administered local anesthetics mainly relies on the flow of blood through the site of its administration/application. Moreover, the inadvertent parenteral injection of an adjuvant local anesthetic combination would be much safer because the low concentrations of local anesthetic would be less likely to cause life-threatening events such as seizures, respiratory paralysis, or myocardial depression (55).

The addition of vasoconstrictors to local anesthetic formulations is shown to increase their analgesic effects (56). Clonidine, when given in combination, is shown to prolong the analgesic effect of local anesthetics like lidocaine (mean duration of 770 min) in axillary brachial plexus block (23). It was found that a small dose of clonidine (between 30 and 90 µg) was able to increase the quality of peripheral nerve block from lidocaine with potentially lower risk of an alpha-2 receptor agonist side effects of sedation. Adding epinephrine to lidocaine solution was also found to increase the intensity and duration of sciatic nerve block in the rat (24). These vasoconstrictors are believed to decrease systemic absorption of local anesthetics thereby increasing their local concentration (56).

Ibutilide, a class III antiarrhythmic methane sulfonanilides, significantly increases bupivacaine's local anesthetic potency by 2.6-fold. Though it has no analgesic effect when given alone, ibutilide is supposed to bind at a similar channel, sodium ion channels, with local anesthetics because it contains an amide-link characteristic of local anesthetics. Co-administration of ibutilide with bupivacaine and epinephrine combination further increased the potency of bupivacaine another 6.8-fold beyond the 2.3-fold enhancement elicited by the addition of epinephrine (57).

There are also reports of prolonged analgesia upon the addition of corticosteroids as an adjuvant to local anesthetics. Though the exact mechanism of action of corticosteroids is not clearly understood, inhibition of inflammatory mediators (58) and their vasoconstriction effect when applied topically (59) are expected to play a role. Inflammatory mediators involved in the acute phase response such as tumor necrosis factor-alpha (TNF-a), interleukins (IL-1 b, IL-6, IL-8, and others), and prostaglandin (PGE2) are known to stimulate nociceptors thereby increasing pain. According to Movafegh et al. (60), the addition of dexamethasone to lidocaine 1.5% solution in axillary brachial plexus block prolongs the duration of sensory (242 min vs. 98 min in control) and motor (310 min vs. 130 min in control) blockade. Dexamethasone, when given in combination, also prolongs the analgesic effect of bupivacaine by 1.75-fold (61) and reduces the need for opioid use. Despite its narrow margin of safety, co-encapsulation of dexamethasone, bupivacaine, and tetrodotoxin was also reported to produce prolonged local analgesia (62).

Another study (63) showed that combined administration of local anesthetics and CaCl_2_ results in a significant prolongation of lidocaine and bupivacaine effects with the mechanism supposed to be due to a raised threshold for nerve excitation is unlikely to become clinically useful as an adjuvant for prolonged local analgesia. Nonetheless, this formulation is unlikely to become clinically useful for prolonged local analgesia since the addition of calcium, especially at high concentrations, to local anesthetics has significant neurotoxicity.

#### 2.3.2 Liposomal formulations

Liposomes (lipid vesicles) are sealed sacs in the micron or submicron range dispersed in an aqueous environment. The walls of the sac consist of bilayers composed of suitable lipids. The nature of the bilayers allows the formation of an internal aqueous compartment. Local anesthetics can be loaded into either the aqueous or lipid phases for later release after being injected into biological tissue (27, 64).

According to a study done by Boogaerts et al. (25), the duration of analgesia of bupivacaine was increased from 3.2 h with the plain solution to 6.25 h with the liposomal preparation. This study also indicated that a significant prolongation of analgesia was observed in patients receiving an epidural injection of liposomal anesthetic (from 2.42 to 10.6 h) compared with plain 0.5% local anesthetic (2.4 h) solution after abdominal aortic surgery. Moreover, no motor block was seen in those subjects with the liposomal preparation indicating that this concentration (0.5%) of liposomal bupivacaine can be used for postsurgical analgesia with an increased duration of action and lower interference with patient functionality. A retrospective cohort study done on patients who had undergone total hip arthroplasty reported that the use of liposomal bupivacaine resulted in a decreased need for opioid use within 24 h postoperatively and decreased length of stay requirements from 2.47 days to 1.93 days (26).

Ropivacaine hydrochloride multivesicular liposomal formulation also demonstrated significantly sustained release durations both *in vitro* and *in vivo* compared with both ropivacaine liposomal and ropivacaine hydrochloride free solutions (27). Another study done in rats by Mcalvin et al. (65) showed that the duration of sensory block achieved by multivesicular liposomal bupivacaine (Exparel®) was approximately twice that achieved with a commonly used concentration of bupivacaine HCl (0.5% w/v). This result is strengthened by another study (22) which stated that wound infiltration of multivesicular liposomal bupivacaine imparts a longer duration of postoperative pain relief compared to plain bupivacaine. Additionally, this study reported that an opioid-sparing effect, higher patient satisfaction, earlier discharge, and lower hospital costs with achieved with the use of Exparel®. A recent study also supports this finding which reports that liposomal suspension of bupivacaine demonstrated an effective anesthetic block during castration which is comparable with a multimodal approach of lidocaine and meloxicam (66).

However, Schroer et al. (67) reported a result that contradicts the previously discussed articles. It was a prospective study done on patients undergoing total knee arthroplasty which showed that multivesicular liposomal bupivacaine did not demonstrate improved pain scores, lower narcotic use, or better knee motion during hospitalization. However this study has some limitations; first, the surgeon was not blinded at the time of the injection and secondly, liposomal bupivacaine is a cloudy liquid that is more viscous and therefore harder to inject than clear bupivacaine.

#### 2.3.3 Lipid-based nanoparticles

Lipid-polymer hybrid nanoparticles (LPNs) are another novel class of therapeutic delivery vehicles that have excellent stability with storage and controlled release, in contrast to liposomes, which significantly leak medication during prolonged storage at 4°C. The two primary components of LPNs are polymer cores and one or more lipid layers that make up the shells. The lipid shells (the outside components) cover the polymer core's exterior surface and act as barriers to stop medications from leaking out quickly while permitting a slow, controlled release. Both hydrophilic and hydrophobic pharmaceuticals can be enclosed in the polymer cores (the inner sections), which are made up of poly (lactic-co-glycolic acid) (PLGA), poly (beta-amino ester), dextran, etc. (29).

Moreover, LPNs combine the mechanical advantages of biodegradable polymeric nanoparticles and the biomimetic advantages of phospholipids including high drug loading and good serum stability. Hence, the use of nanoliposome (nanometric version of liposomes) formulations of local anesthetics will help achieve greater effectiveness, increased safety, reduced likelihood of toxicity, and decreased side effects which is a breakthrough in medical practice and a great advantage for the safety and comfort of the patient (21, 29).

Chitosan and hyaluronic acid-modified layer-by-layer lipid nanoparticles of lidocaine showed a longer anesthetic effect (that persisted for 60 min after the application) than the lidocaine solution formulation (21). Such nanoparticle formulations provide a prolonged release of the loaded anesthetic agents. These formulations generally have revealed a more interesting rapid anesthetic effect in the first few minutes, and sustained activity compared with the other formulations. A long-lasting (36 h) analgesic effect was also reported with ropivacaine-loaded LPNs (28).

Another study (29) reported that bupivacaine lipid-polymer hybrid nanoparticles exhibited prolonged *in vitro* release in phosphate-buffered saline (pH = 7.4), enhanced *in vitro* stability in 10% fetal bovine serum, and lower cytotoxicity compared with bupivacaine-loaded PLGA nanoparticles. In addition, bupivacaine LPNs exhibited significantly prolonged analgesic duration than bupivacaine nanoparticles (30).

#### 2.3.4 Polymeric matrices

Biodegradable polymers are used to prepare matrix (monolithic) systems in which the drug is dispersed or dissolved homogeneously throughout the polymer (38). A major advantage of a biodegradable polymeric-controlled drug delivery system over others is that it does not require the surgical removal of the drug-depleted device. Common drug delivery systems such as polylactic acid polymers display bulk erosion and could release potentially toxic amounts of the drug *in vivo*. Whereas newer polyanhydride polymer-drug matrices erode primarily from the surface, and hence drug is released to the surrounding solution as layers of polymer are eroded from the surface. Release characteristics of polymers can be adjusted by altering the composition of the polyanhydride matrix to the desired lipophilicity and hydrophilicity (32, 68).

Another study showed that analgesic-loaded microparticles possessed low toxicity against human fibroblasts and were able to sustainably elute levobupivacaine, lidocaine, and acemetacin *in vitro*. Such formulations were also found to release high levels of lidocaine and acemetacin, and levobupivacaine at the fracture site of rats for more than 28 days and 12 days, respectively (31).

*In vivo* experiments involving the implantation of polymer local anesthetic matrix devices, loaded with 20% bupivacaine through hot melt incorporation, resulted in a reversible sciatic nerve blockade lasting for four days when implanted adjacent to the sciatic nerve of rats (32). Perisciatic nerve injection of PLGA-coated ropivacaine showed an analgesic effect persisting for about a week (33). Ropivacaine and dexamethasone-loaded PLGA microparticles via electrospraying technique showed high concentrations of ropivacaine and dexamethasone at the target region *in vivo* for over two weeks while the drug levels in the blood remained low (34).

#### 2.3.5 Thermo-responsive gels

A group of biomaterials known as thermogels can function as injectable solutions at room temperature and transform into colloidal gels on-site as they warm to body temperature. Since the medication may be easily dissolved and then injected into the patient, whereupon the thermogel will continue drug delivery at the injection site, these materials are excellent for prolonged, localized anesthetic delivery. A range of different synthetic or natural polymers can be used to produce thermogels (38). The use of such agents for the preparation of extended-release products further advanced the previous reports on the use of various gel formulations of local anesthetics.

It was shown that a prolonged duration of release was observed from a 2% lidocaine hydrochloride gel formulated with four different polymers; methylcellulose, hydroxyl propyl methyl cellulose, sodium carboxy methyl cellulose, and poloxamer 407. Among these, poloxamer exhibited the slowest release (240 min) while methylcellulose showed the fastest release (90 min) (35). A similar prolonged analgesic effect was reported by Wang et al. (36) after the implantation of a controlled-release delivery system containing 16% (w/w) lidocaine next to the sciatic nerve of male rats.

Another study (16) also reported that significantly prolonged analgesia was achieved in the case of lidocaine when poloxamer gel (25%) containing 2% lidocaine HCl or 2% ibuprofen sodium was administered epidurally to pigs. The poloxamer gel preparation resulted in reduced systemic absorption of both drugs but increased epidural availability only in case of lidocaine. This result is comparable with a report of a later study (37) where prolonged release of lidocaine over 48 h was observed from a combination of lidocaine and poloxamers, P407 and P188. Ropivacaine prepared with P407/188 was also reported to have lower *in-vitro* cytotoxicity, increased duration of analgesia, and no signs of *in vivo* inflammation (39).

Duration of analgesia and extent of local inflammatory response of thermosresponsive nanogels were found to be dependent on the size of local anesthetic gel formulations. Small (<300 nm) acid-functionalized poly(N-isopropylacrylamide)-based bupivacaine nano gels resulted in durations of sciatic nerve blockade of up to 8–9 h while inducing only a mild inflammatory response (69). Whereas, large (800–1,000 nm) acid-functionalized nanogels provided moderate durations of nerve block (5–6 h). They also induced an extensive inflammatory response in which a thick inflammatory capsule formed around the injected nanogel suspension. Fu et al. (70) reported that the analgesic effect of a single injection of ropivacaine-loaded PLGA thermo-responsive gel at the incision site lasted for 48 h, which is significantly longer than the effect produced by injection of ropivacaine solution alone (almost 2 h). This strengthens the results of a previous study done in rats (51), which reported that a single treatment with lidocaine-loaded slow-release lidocaine sheet (SRLS) with PLGA inhibited hyperalgesia and c-fos (an immune reactive antibody) expression in the spinal cord dorsal horn for 1 week.

Another approach is to prepare thermo-responsive nanogel of local anesthetics with chitosan that has shown promise as an injectable drug delivery vehicle for over ten years. Ropivacaine base nanoparticles, fabricated and entrapped with dexamethasone using a chitosan thermogel controlled release system, demonstrated sustained analgesia for up to 48 h *in vivo* (38). The inclusion of a small dose of dexamethasone was also reported to further improve the analgesic efficacy of ropivacaine to a large content (71). Furthermore, Benzocaine-loaded PLGA nanoparticles were also shown to be a promising drug delivery system for LAs, prolonging anesthetic efficacy, and decreasing toxicity (40).

#### 2.3.6 Microemulsions, microspheres and microcrystals

Microspheres provide sustained release in localized areas and can be employed to reduce total required medication doses and frequency of use. Drug release is affected by the physical structure and chemical properties of the microsphere and encapsulated drug. Polymer molecular weight, blend composition, type of polymer and drug crystallinity, drug distribution, sphere porosity, and sphere size are the major factors found to influence the release profile and should be tailored to fit a desired release. Degradation of the microcapsule polymer and diffusion of the drug through the pores of the capsule were the major determinants of the rate of drug release from the device (68). The biocompatibility of microspheres is achieved with the use of naturally occurring polymers and monomers such as cellulose and glycolic acid.

Based on an *in vitro* study (41), sustained release of dibucaine was achieved from polylactic acid (PLA) microspheres and the local anesthetic effect of this preparation was also found to be prolonged (300 h).

Tetracaine (10%) lecithin-coated microcapsules resulted in prolonged duration (lasting 43.4 h) reversible anesthesia whereas plain solution of similar concentration of tetracaine produced death in 60% of animals. Moreover, survivors experienced wet gangrene of the tails, with a mean tail nerve block duration of only 8.5 h (42). This result indicates that the microencapsulated formulation releases small portions of the drug over an extended period while the plain solution releases its content almost immediately and demonstrates a higher extent of absorption.

Curley et al. (43) developed a bupivacaine polyester microsphere local anesthetic injection, which provides 2–5-day blockage of the sciatic nerves of rats *in vivo*. Bupivacaine microspheres are shown to be safe and effective means for producing intercostal nerve blocks in a large animal (sheep), representing large species comparable with an adult human in both body weight and length of nerves. The incorporation of dexamethasone into bupivacaine microspheres also resulted in significantly prolonged nerve blockade (44).

Co-encapsulation of tetrodotoxin, a naturally occurring sodium channel blocker with very potent local anesthetic properties, in controlled release devices containing bupivacaine and dexamethasone, resulted in very prolonged nerve blocks (median nociceptive block duration of 221.7 h). However, results from this study (62) showed that the preparation has a narrow margin of safety and the probability of this formulation being incorporated into clinical practice is unlikely.

*In vivo* studies of lidocaine microspheres, prepared by the o/w emulsion technique using PLGA, in rats showed that the area under the plasma level curve (AUC) of lidocaine in microspheres was 2.02–2.06-fold that of conventional lidocaine solution injection. Despite there being significant dose dependency, pharmacodynamics results also showed that lidocaine microspheres showed a significant increase in the duration of release of the medicament (72).

Extended duration formulation of 15% bupivacaine in poly (DL-lactic acid co-castor oil) synthesized by ring-opening polymerization resulted in prolonged duration of local anesthesia effect. However, no significant differences in mechanical withdrawal response by the Von Frey test were observed in the animal model up to at least 48 h (73).

#### 2.3.7 Multivalent-ion complexation

A complex formed by an ionized drug and a multivalent counter-ion can be formed that offers a sustained release of the drug without the need for an additional delivery matrix (74). Since local anesthetics are weakly basic compounds, they are positively charged in aqueous solutions; hence can be complexed with negatively charged ions.

Lidocaine/multivalent ion complex was prepared and its release profile was studied through *in vitro* and *in vivo* experiments (3). Lidocaine, a positive ion in aqueous media, was mixed with K_3_PO_4_ which gives the anion PO_4_^3−^ in water to form a multiple ion lidocaine complex. After a mild initial burst of lidocaine release (15%) for 1 h, the ion complexed lidocaine continuously released lidocaine at a constant rate (4%/h) for 24 h and release was almost complete. However, the duration of sciatic nerve blockade was found to be dose-dependent; with the high dose (complex containing 100 mg of lidocaine) showing dramatically prolonged (14 h) nerve block as compared to that of the low dose (complex containing 10 mg lidocaine) which has less than 2 h. This could probably be inferred to the sustained release of lidocaine from the complex at a sufficient concentration to achieve anesthesia. Nonetheless, it is better to use other salts to ameliorate the potential of hyperkalemia from the lidocaine/ion complex will be of great concern.

#### 2.3.8 Complexation with cyclodextrin

Cyclodextrins are among the most promising carriers for the sustained release of anti-nociceptive agents from which hydroxypropyl-betacyclodextrin (HP-β-CD) has been approved for parenteral use. HP-β-CD is well tolerated in humans and, after intravenous administration, is almost completely eliminated via glomerular filtration (75). Owing to its hydrophilic nature, HP-β-CD cannot easily diffuse across membranes thereby exhibiting slow absorption into the systemic circulation. Inclusion of the drug into cyclodextrin is reported to bring about prolonged release for different local anesthetics including tetracaine (45), a combination of bupivacaine and clonidine (46), benzocaine (49), ropivacaine (46), and lidocaine (50).

The intensity and duration of analgesia from local anesthetics could further be enhanced by entrapment of the drug-HP-β-CD complex into liposomes. This was demonstrated by an *in vivo* study done on rabbits which reported that benzocaine elicited a significantly improved intensity and duration of anesthesia when benzocaine-HP-β-CD is loaded in multi-lamellar vesicles (MLVs) compared with benzocaine MLVs (49). Domingues et al. (76) also reported that bupivacaine complexed in sulfobutylether-β-cyclodextrin had a significantly prolonged (about 2 h) antinociceptive effect compared to plain bupivacaine.

The safety aspect of these type of formulations was also studied by a separate study (47) which indicated that ropivacaine-MLV led to an increased release of all pro-inflammatory cytokines (IL-1a TNF-a, IL-6, and IL-10), and the HP-β-CD form was a better drug carrier than the MLV form since it increases only IL-6 by two-fold. Another previous study also reported that HP-β-CD forms of bupivacaine and ropivacaine showed lower myotoxicity and similar cytotoxic effects when compared to their corresponding plain solutions (48).

#### 2.3.9 Bio-adhesive films

Another strategy to prolong regional analgesia is through bio-adhesion, which can increase the amount of time the formulation is in touch with the anesthetic surface. Fast-acting and long-lasting bioadhesive films of benzocaine (3% or 5%) with the proper proportions of the penetration enhancer, a combination of propylene glycol and Transcutol, were more effective with no local toxicity in comparison to commercial semisolid formulations containing the same drug dose (46). A slow-release lidocaine sheet (SRLS) with PLGA was also able to produce a sustained effect for 1 week without inducing inflammation of the sciatic nerve in a rat model (51). It was also reported that lidocaine gel containing diethylene glycol had about a 3.89-fold increase in analgesic activity (52). Carr & Horton (53) and Cho et al. (54) also pointed out that lidocaine and ropivacaine-containing bio-adhesive patches showed higher and prolonged local analgesic effects, respectively.

Another approach is the use of a microneedle-integrated transdermal patch (MITP), which allows prolonged localized analgesia. Lidocaine encapsulated MITP was reported to be a useful alternative to injections and passive transdermal systems with lidocaine permeating skin within 5 min of MITP application. This faster permeation enables a possibly rapid means of relief of pain for patients (51).

### 2.4 Limitations of sustained release formulations

The first problem with these formulations is that they may not be suitable for all patients. For example, the use of vasoconstrictor and corticosteroid adjuvants may be contraindicated in some patient populations. Vasoconstrictors, for instance, may aggravate cardiovascular conditions in patients with hypertension and dysrhythmia. Corticosteroids on the other hand may exacerbate hyperglycemia in diabetic patients and edema in congestive heart failure and/or patients with renal failure, resulting in an increased risk of infection in immunocompromised patients (24).

The other limitation is the issue of poor *in vitro*-*in vivo* correlation. Actual *in vivo* performances of these controlled-release local anesthetics are mostly poorly mirrored by their corresponding *in vitro* characteristics. For instance, surgical analgesia was not obtained when patients were given liposomal local anesthetics. This could be explained by the slow release of the drug from the liposomes, which limited the amount of free anesthetic present at the site of action (25). This is also supported by another prospective study done on patients after total knee arthroplasty, which showed that no significant difference was observed between liposomal bupivacaine and bupivacaine solution (67). Due to their complex production, composition, and release mechanisms, no *in vitro*-*in vivo* correlation guidelines are developed for these controlled-release products (77).

In addition, the length of time that a medicine remains active after being encapsulated frequently outlasts the time that it has a therapeutic effect. For instance, *in vitro* drug release of 50%–75% (w/w) bupivacaine PLGA microspheres continued for more than 40 days while generating sensory nerve blockade lasting fewer than 12 h (62). As a result, drug release lasts for a long time but is insufficient to reach clinically useful concentrations.

Because biodegradable polymers are chemically unstable, their use as reservoir delivery systems is potentially hazardous. The potential for these polymers to degrade prematurely thereby releasing the remaining contents of the drug reservoir presents a safety concern. If this happens, potentially toxic levels of the local anesthetic will reach systemic circulation. Allergy and inflammation of the skin due to the application of transdermal patches containing local anesthetics may also become an issue in some patients (51).

## 3 Conclusions

The duration of analgesic release from many of the formulations discussed in this review is far longer than the duration of analgesia. Additional research efforts are required to manage this situation to minimize the risk of adverse events. Further safety studies are required to ensure the safe and effective utilization of sustained-release local anesthetics. Moreover, the release kinetics of the various formulations should be adequately established.
